# Negative association between acrylamide exposure and body composition in adults: NHANES, 2003**–**2004

**DOI:** 10.1038/nutd.2016.48

**Published:** 2017-03-13

**Authors:** P-L Chu, L-Y Lin, P-C Chen, T-C Su, C-Y Lin

**Affiliations:** 1 Department of Internal Medicine, Hsinchu Cathay General Hospital, Hsinchu, Taiwan; 2 Graduate Institute of Biomedical and Pharmaceutical Science, College of Medicine, Fu Jen Catholic University, New Taipei City, Taiwan; 3 Department of Internal Medicine and Cardiovascular Center, National Taiwan University Hospital, Taipei, Taiwan; 4 Institute of Occupational Medicine and Industrial Hygiene, College of Public Health, National Taiwan University, Taipei, Taiwan; 5 Department of Public Health, College of Public Health, National Taiwan University, Taipei, Taiwan; 6 Department of Environmental and Occupational Medicine, National Taiwan University College of Medicine and National Taiwan University Hospital, Taipei, Taiwan; 7 Department of Internal Medicine, En Chu Kong Hospital, New Taipei City, Taiwan; 8 School of Medicine, Fu Jen Catholic University, New Taipei City, Taiwan

## Abstract

**Background/Objectives:**

Acrylamide is present in mainstream cigarette smoke and in some food prepared at high temperature. Animal studies have shown that acrylamide exposure reduces body weight. Prenatal exposure to acrylamide also has been linked to reduced birth weight in human. Whether acrylamide exposure is associated with altered body compositions in adults is not clear.

**Subjects/Methods:**

We selected 3623 subjects (aged ⩾20 years) from a National Health and Nutrition Examination Survey (NHANES) in 2003–2004 to determine the relationship among hemoglobin adducts of acrylamide (HbAA), hemoglobin adducts of glycidamide (HbGA) and body composition (body measures, bioelectrical impedance analysis (BIA), dual energy x-ray absorptiometry (DXA)). Data were adjusted for potential confounding variables.

**Results:**

The geometric means and 95% CI concentrations of HbAA and HbGA were 60.48 (59.32–61.65) pmol/g Hb and 55.64 (54.40–56.92) pmol/g Hb, respectively. After weighting for sampling strategy, we identified that one-unit increase in natural log-HbAA, but not HbGA, was associated with reduction in body measures (body weight, body mass index (BMI), subscapular/triceps skinfold), parameters of BIA (fat-free mass, fat mass, percent body fat, total body water) and parameters of DXA (android fat mass, android percent fat, gynoid fat/lean mass, gynoid percent mass, android to gynoid ratio). Subgroup analysis showed that these associations were more evident in subjects at younger age, male gender, whites, lower education level, active smokers and those with lower BMI.

**Conclusions:**

Higher concentrations of HbAA are associated with a decrease in body composition in the US general population. Further studies are warranted to clarify this association.

## Introduction

Acrylamide is widely used in industry to manufacture numerous products, including adhesives, mining chemicals, fibers, pharmaceuticals, animal feed, paper sizing, molded parts, textiles and coagulant aids, and to prepare polyacrylamide gels for electrophoresis in biological laboratories.^1^ In 1994, acrylamide was classified as a probable human carcinogen by the International Agency for Research on Cancer.^2^ Acrylamide gained a great deal of public and scientific attention when the World Health Organization (WHO) published the concentrations of acrylamide in several food in 2002.^3^ In high temperature-processed carbohydrate-rich food, acrylamide can be generated from variable reactions, particularly between asparagine and glucose.^4, 5^ The WHO has reported an average dietary intake of acrylamide of 0.3–0.8 μg/kg/day, based on several studies.^6^ In addition to dietary exposure, acrylamide is also a major component of cigarette smoke.^7^

In human body, the majority (85%) of acrylamide is conjugated with glutathione, whereas the rest of acrylamide (15%) is activated by cytochrome P-450 CYP2E1 to become a reactive epoxy compound, glycidamide. Glycidamide is subsequently metabolized by hydrolysis and conjugated with glutathione. Acrylamide and glycidamide contain an α,β-unsaturated system that reacts with nucleophilic compounds via a Michael addition. N-(2-Carbamoylethyl) valine and N-(2-Carbamoylehydroxyethyl) valine are formed through reactions of N-terminal valine residue in hemoglobin (Hb) with acrylamide and with glycidamide, respectively.^8^ With constant exposure, a steady level of the corresponding Hb adduct will be reached, which can be used a surrogate of the internal acrylamide level and degree of acrylamide exposure in the past 4 months.^9^ Hemoglobin adducts of acrylamide (HbAA) and glycidamide (HbGA) have been proposed as biomarkers of acrylamide exposure and used to estimate the internal level of acrylamide in the general population.^10, 11, 12, 13^

Experiments in rodents have demonstrated that acrylamide exposure could lead to neurotoxicity, developmental and reproductive toxicity, genotoxicity and carcinogenicity.^14^ In human, the biological consequences of acrylamide exposure have mainly centered on neurotoxicity because this adverse event was observed in people who were occupationally exposed to this compound.^15, 16^ The epidemiological evidence of the relationship between acrylamide and cancer in general population remains inconsistent and ambiguous.^17, 18, 19^ In addition to neurotoxicity and carcinogenicity, acrylamide exposure also has been linked to altered thyroid function,^20^ sex hormones,^21^ increased oxidative stress,^22^ as well as reduced insulin resistance^10^ in epidemiological studies.

In animal studies, reduction in offspring body weight following maternal acrylamide exposure during gestation had been consistently observed in mice and rats.^23, 24^ The body weight-lowing effect of acrylamide was repeatedly demonstrated in many animal models in either acute,^25, 26^ intermediate^25, 27, 28^ or chronic exposure.^25^ It has been shown that daily high dose acrylamide exposure can result in a decrease in appetite motivation or a reduced rate of weight gain,^29, 30^ which might be mediated through increased oxidative stress.^26^ In human, a large population-based study in Europe has provided the epidemiological evidence showing a significant association between prenatal exposure to acrylamide and reduction in birth weight as well as head circumference.^13^ Moreover, in cross-sectional studies, HbAA has been found to be inversely associated with body mass index (BMI) in smokers.^10, 31^ If the association between acrylamide and body weight is real, although further study is needed, the alteration in body composition after acrylamide exposure represents an interesting public health issue. The goal of this present study is to determine the association between acrylamide exposure and body measurements by examining data from the National Health and Nutrition Examination Survey (NHANES) that was collected from 2003 to 2004.

## Materials and methods

### Study design and population

Data were acquired from the 2003 to 2004 NHANES. The NHANES, a population-based survey, is designed to collect information regarding the health and nutrition in U.S. households and to obtain a representative sample of the non-institutionalized civilian U.S. population. The survey data are published biannually. The complete contents of the NHANES 2003–2004 are available online at the NHANES website.^32^ The analyses were limited to 4152 participants who were at least 20 years of age and had been tested for HbAA and HbGA. From these participants, we conducted further analyses on 3623 subjects without missing data on demographics, smoking status and BMI.

### Assessment of HbAA and HbGA

In the NHANES, whole blood or erythrocytes were used to measure HbAA and HbGA. Specifically, the reaction products containing N-terminal valine of the Hb protein chains (N-(2-carbamoylethyl) valine and N-(2-hydroxycarbamoyl-ethyl) valine for acrylamide and glycidamide adducts, respectively) were measured. This measurement method is derived from the modified Edman reaction that measures the ability of N-alkylated amino acids to form Edman products in neutral or alkaline conditions rather than the acidic conditions required in conventional Edman reaction.^33^ Quantitation of HbAA and HbGA was performed using octapeptides with identical amino acid sequence to the N-terminal of the Hb beta-chain, acrylamide and glycidamide attached at the valine (AA-VHLTPEEK, GA-VHLTPEEK). The corresponding stable isotope-labeled AA-Val (13C5 15N)-HLTPEEK serves as the internal standard. Total Hb measurement was performed using calibrators provided with the manufacturer's assay kit. The detection thresholds were 2 pmol/G Hb for HbAA and 3 pmol/G Hb for HbGA. If the concentrations were below the detection thresholds (1.8% of blood samples for HbGA), a value was assigned by NHANES, and this value was used in our analyses. Detailed information is available at the NHANES website.^34^

### Body measures

The body measurement assessments of survey participants varied based on participants' ages. Body weight data of individuals who had limb amputed were excluded. This data set includes body measurements of women who were pregnant at the time of the exam. BMI was derived from the body weight (in kilograms) divided by body height (in meters square). The NHANES anthropometry protocol was employed to measure both triceps and subscapular skinfold (in millimeters) in participants older than 2 months using the Holtain skinfold caliper (Holtain, UK). The techniques of body measurement were illustrated in the NHANES III Anthropometric Procedures Video. Further procedures relevant to this component are available at the NHANES website.^35^

### Dual energy X-ray absorptiometry (DXA)—android/gynoid

The DXA is the most widely accepted method used in body composition measurement because of its speed, ease of use, and low radiation.^36^ The radiation exposure from whole body DXA scans is extremely low that is less than 10 uSv. In the 2003–2004 NHANES database, whole body DXA scans were performed in eligible participants that are at least eight year-old. The exclusion criteria of the DXA examination included pregnancy, and any self-reported history of radiographic contrast material (barium) use in the past seven days, nuclear medicine studies in the past three days, or body figure is not allowed to fit the DXA table (weight>300 pounds or height>6′5″). The DXA examinations were conducted by certified radiology technologists.

Briefly, the whole body DXA scans were acquired using the Hologic QDR-4500A fan-beam densitometer and its software (version 8.26:a3*) (Hologic, USA). The DXA technique acquires low-dose X-ray images from two different average energies. The attenuation ratio of these two average energies, the R-factor, can be used to distinguish bone from soft tissue, and the percentage of fat in soft tissue when bone was subtracted. The android and gynoid regions were defined by the Hologic APEX software used in the scan analysis. The android area is roughly the area around the waist between the mid-point of the lumbar spine and the top of the pelvis, whereas the gynoid area lies roughly between the head of the femur and the mid-thigh. Whole body scan analyses allow soft tissue measurements of the android and gynoid areas and android/gynoid ratios. More details of the DXA examination protocol are available on the NHANES website.^37^

### Bioelectrical impedance analysis (BIA)

Bioelectrical impedance analysis, the measurement of electrical impedance of body tissues, is used to measure total body water, fat mass as well as fat-free body mass. This examination was conducted in eligible survey participants aged eight to 49 years. Pregnant females were excluded from this BIA measurement. Those who have amputations other than fingers or toes, implants such as artificial joints, pins, plates or other types of metal objects in the body, pacemakers, automatic defibrillators, coronary stents, metal suture material in the heart or body weight greater than 300 pounds (limitation of the examination table) were excluded from the analysis.

Briefly, the NHANES BIA results were obtained using the HYDRA ECF/ICF Bio-Impedance Spectrum Analyzer (Model 4200, Xitron Technologies, USA). The multi-frequency analyzer employs a full 12-bit digital signal processing technique to measure impedance at 50 frequencies logarithmically spaced between 5 KHz and 1 MHz. While the alternating current passed through surface electrodes placed on the right hand and foot, the impedance to the current flow was measured by other electrodes adjacent to the injection electrodes. The voltage drop between electrodes serves as an indicator of impedance, or opposition to the flow of the electric current. More information is available at the NHANES website.^38^

### Covariates

We considered age, gender, race/ethnicity, education level, household income, smoking status, caffeine intake, total energy intake, total protein intake, total sugar intake, total carbohydrate intake, total saturated fatty acid intake, metabolic equivalent intensity level for activity as potential confounders of body composition in our analyses. Data were collected at all study sites by trained personnel using standardized procedures. Sociodemographic information, such as age, gender, race/ethnicity, education level and household income, was recorded during the household interview. Smoking status was categorized as active smoker, exposed to environmental tobacco smoke, or non-exposed by the smoking questionnaire and serum cotinine levels as described previously.^39^ Serum cotinine was measured by isotope dilution-high performance liquid chromatography/atmospheric pressure chemical ionization tandem mass spectrometry. Active smokers were defined as those with cotinine levels>15 ng/ml or those who reported currently smoking every day or on some days. Those with serum cotinine levels that were detectable but ⩽15 ng/ml and who did not report current smoking were considered as exposed to environmental tobacco smoke. Cotinine levels of<0.015 ng/ml were below the detection limit. Those with undetectable serum cotinine levels, no reported smoking at home, and no self-reported smoking were considered as non-exposed.

A two-day dietary intake data from each participant were used to estimate the types and amounts of foods/beverages consumed during the 24-h period before the interview (midnight to midnight), and the intake of energy, nutrients, and other food components from those foods and beverages. The first day data were collected in the Mobile Examination Center, whereas the second day data were collected over telephone 3 to 10 days later. The caffeine intake, total energy intake, total protein intake, total sugar intake, total carbohydrate intake and total saturated fatty acid intake from the two days were averaged as covariates in this study. All participants older than 12 years were eligible for information about specific leisure-time activities. Metabolic equivalent scores for the activities were obtained from the appropriate reference and through personal communication with the author.

### Statistics

HbAA and HbGA concentrations were expressed as the geometric mean with a 95% confidence interval (CI) in different subgroups, and tested by the Student's 2-tailed *t*-test as well as one-way analysis of variance (ANOVA). Because of the significant deviation from the normal distribution, natural log-transformation of HbAA and HbGA was adapted. We later used body components as a dependent variable and individual natural log-transformed HbAA and HbGA as a predictor in an extended model analysis. Model 1 adjusted for age (continuous variable), gender (categorical), race and ethnicity (categorical). Model 2 adjusted for model 1 plus education level (categorical), household income (categorical), smoking status (categorical), caffeine intake (continuous variable), total energy intake (continuous variable), total protein intake (continuous variable), total sugar intake (continuous variable), total carbohydrate intake (continuous variable), total saturated fatty acid intake (continuous variable) and metabolic equivalent intensity level for activity (continuous variable). To avoid model-dependent association, an association was considered significant only when it remained statistically significant in all models. To assess the dose-response effects across the population, HbAA and HbGA were further stratified across the population in quartiles. Analyses were performed using sampling weights to examine the effects of weighting. Sampling weights were derived using procedures based on the National Center for Health Statistics analytic guidelines^40^ and properly accounted for the complex survey design of the NHANES 2003–2004. Sampling weights accounting for unequal probabilities of selection, oversampling and nonresponse were applied to all analyses using the complex sample survey module of SPSS Version 20 for Windows 7 (SPSS, USA). *P*<0.05 was considered significant.

## Results

The study enrolled 1753 men and 1870 women, and the basic demographics of the sample population are outlined in Table 1. The HbAA and HbGA were detectable in 100% and 98.2% of study subjects, respectively. The median concentrations (25th and 75th percentile) of HbAA and HbGA were 53.4 (40.9–80.1) pmol/g Hb and 55.4 (39.5–81.3) pmol/g Hb. The results indicate that younger age, non-hispanic black respondents, high school education level, active smoker (cotinine levels>15 ng/ml or those who reported currently smoking every day or on some days), higher caffeine and saturated fatty acid intake were associated with higher HbAA and HbGA concentrations. Specifically, male participants and those with BMI below 25 had a higher concentration of HbAA.

The linear associations between HbAA levels and various body component measures in sample subjects weighted for sampling strategy are shown in Table 2. In addition to android lean mass, the HbAA level was inversely associated with almost all measures of body components, including weight, BMI, waist, subscapular skinfold, triceps skinfold, estimated fat-free mass, estimated fat mass, estimated percent body fat, estimated total body water, android fat mass, android percent fat, gynoid fat mass, gynoid lean mass, gynoid percent fat and android to gynoid ratio. Interestingly, there were no significant associations between the HbGA level and body components measures.

To evaluate the dose-response relationship, the HbAA and HbGA were stratified across the population in quartiles in Table 3. After adjustment of potential confounders by regression analyses, the results showed that body weight and BMI significantly decreased across quartiles of HbAA concentrations from 4th quartile (Q4,>80.10 pmol/g Hb) to the first quartile (Q1, ⩽40.90 pmol/g Hb) (Q4 vs Q1, *P*=0.005 and 0.003, for body weight and BMI, respectively). Estimated fat mass and android fat mass also significantly decreased across quartiles of HbAA concentrations from the fourth quartile to the first quartile (Q4 vs Q1, *P*=0.008 and 0.002, respectively). The results of subgroup analyses were demonstrated in Table 4. The associations between two body composition measures (BMI and android fat mass) and HbAA levels were more evident in individuals at younger age (20–39 year-old), male gender, white ethnic, a lower education level, active smoking and lower BMI.

We also investigated the association between HbAA, body composition measures and serum cotinine levels in active smokers in Table 5. The associations between HbAA and body composition parameters including weight, BMI, estimated fat mass and gynoid lean mass were more evident in individuals with higher serum cotinine levels, whereas estimated percent body fat, and gynoid percent fat were more evident with lower serum cotinine levels. The association between HbAA and other parameters of body composition were not related to serum cotinine levels in active smokers.

## Discussion

To our knowledge, this is the first study to show an inverse association between HbAA level and body composition measures in a nationally representative survey of US adults. The main strength of this study is its representative study population.

We report a median concentration (25th and 75th percentile) of HbAA and HbGA of 53.4 (40.9–80.1) pmol/g Hb and 55.4 (39.5–81.3) pmol/g Hb in this study. Our finding is higher than those measured in non-smoking postmenopausal women^11^ and a mother-child cohort in Europe.^13^ However, this concentration is slightly lower than that reported previously in the biomonitoring literature for HbAA levels in an ovarian cancer cohort.^12^ The discrepancy in HbAA and HbGA concentrations between those studies and ours might result from several reasons such as ethnic background, method of measurement, composition of study cohort, definition of smoking and probably life (food) styles, as well as geographic characteristics.

In animal studies, an acrylamide dose of 18 mg/kg/day in male rats resulted in a loss of appetite and a reduced rate of weight gain.^30^ Moreover, daily acrylamide exposure at 5.0 mg/kg/day leads to measurable decrements in aspects of food-motivated behavior in adolescent rats.^29^ Thus, acrylamide exposure might have the potential to produce cognitive or motivational alterations in rats. However, whether the body weight-lowering effect of acrylamide was attributed to the loss of appetite *per se* was questioned in one recent animal study using male Wistar rats which were fed for 12 weeks with acrylamide generated from frying oil heated at 180 °C for 20 h. The amount of ingested acrylamide was estimated to be fairly similar to that of daily human intake (0.28 ppm). The animals fed with acrylamide had a slower rate of body weight increase compared to the control group even though the amount of food ingested did not differ between groups.^41^

In human beings, a European multicenter prospective study has demonstrated an inverse relationship between birth weight and the levels of acrylamide adducts from cord blood.^13^ In our study, the negative association between HbAA and body composition appeared to be independent of smoking status, caffeine intake, total energy intake, total protein intake, total sugar intake, total carbohydrate intake, total saturated fatty acid intake and metabolic equivalent intensity level for activity. The biochemical mechanisms underlying the relationship between low dose acrylamide exposure and reduced body weight without affecting appetite in animals or humans are not clear, but a high pro-inflammatory state such as oxidative stress might be one of the explanations.^26^ Higher doses of acrylamide have also been shown to induce oxidative stress in *in vitro* studies^42, 43^ and in rodents.^44, 45^ In human, chronic ingestion of acrylamide-containing potato chips was found to induce a pro-inflammatory state in 14 healthy volunteers.^46^ In our previous study, we found that urinary acrylamide metabolite concentrations were positively associated with the oxidative stress product in adolescents and young adults.^22^ The ability of acrylamide to readily react with sulfhydryl and amino residues in proteins, including enzymes, receptors, and cytoskeletal proteins, can affect a multitude of cellular processes which has been suggested to form the basis of some of acrylamide's toxic effects^47^ and might contribute to the associations observed in our study.

Since acrylamide exposure might affect thyroid function, it is plausible that the altered thyroid function to influence body weight. However, acrylamide exposure is associated with hypothyroidism, which leads to body weight gain instead of body weight loss.^20^ Thus, the body weight reduction after acrylamide exposure is less likely contributed by altered thyroid hormone. Alternatively, the body weight reduction observed in our study might be induced by acrylamide-mediated sex hormone alteration. The sex hormone levels vary based on the status of menopause, smoking and body weight.^48^ However, Nagata C *et al.* has reported that acrylamide intake was significantly inversely associated with total and free estradiol levels and significantly positively associated with follicle-stimulating hormone level.^21^ Furthermore, it has been reported that estradiol attenuates body weight in monkeys and rodents.^49, 50^ Additionally, the estrogen replacement therapy has been shown to blunt the increases in body weight and adiposity. Similarly, follicle-stimulating hormone, positively associated with acrylamide exposure, is able to increase body weight that is opposite to what we observed in this study.^51^ Collectively, low estradiol, and high follicle-stimulating hormone related to acrylamide exposure might lead to subsequent body weight increase rather than body weight loss. Thus, the body weight-lowering effect observed in our study is less likely result from altered sex hormone.

In the European multicenter study mentioned above, the investigators found an association between elevated levels of acrylamide adducts and reduced birth weights. Moreover, when compared the highest and lowest quartiles (>21.7 pmol/g Hb vs ⩽10.9 pmol/g Hb) of children with prenatal acrylamide exposure from maternal diet, the HbAA was associated with an average birth weight reduction of 157 g, which is 4.5% of the average birth weight of the 1st quartile.^13^ In our study, the mean body weight, average 5.4 kg, decreased significantly with HbAA concentrations from the 4th quartile to the 1st quartile (>80.1 pmol/g Hb vs ⩽40.9 pmol/g Hb). It is 6.6% of the average body weight of the first quartile. The discrepancy in body weight reduction between the European study and ours might be explained by unique basic characteristics of the populations such as race, age (infant vs adults), and age-related characteristics. Moreover, the subjects in our study had higher HbAA levels compared with those in the European study, and this might also contribute to the differences in body weight reduction between two studies. Thus, if the association between HbAA and body weight stands true, it might imply that there is a dose-response relationship.

Android obesity is often referred to as the ‘apple-shape' body figure because the fat is accumulated in the trunk, whereas the gynoid obesity is referred to as the ‘pear-shape' body figure that fat is accumulated in the hip and thigh areas. Fat deposition in the android region is associated with increased risk of cardiovascular diseases, hypertension, hyperlipidemia, insulin resistance, and type 2 diabetes, whereas gynoid fat deposition is associated with decreased risk of metabolic and cardiovascular diseases.^52, 53^ In our previous report using the same study population from the NHANES, the HbAA levels in adults were associated with both reduced serum insulin and insulin resistance.^10^ This phenomenon was also observed in rats.^41^ Thus, the negative association between HbAA level and various parameters of dual energy x-ray absorptiometry, especially fat deposition (android fat mass, android percent fat, gynoid fat/lean mass, gynoid percent mass, android to gynoid ratio) identified in this study might be a potential mechanism leading to the inverse relationship between acrylamide level and insulins resistance.^10^

The association between HbAA and body composition remains significant after we adjusted smoking status, defined by serum cotinine levels or those who reported currently smoking, as confounders. Interestingly, we further identified that the inverse association was more evident among active smokers. In active smokers, we also demonstrated that the association between HbAA and the majority of body composition measures were not change at different serum cotinine levels. Acrylamide is one of the components of cigarette smoke, and its content in mainstream cigarette smoke has been estimated to be 1.1–2.34 μg per cigarette,^7^ which is clearly an important source of acrylamide exposure. The relationship between smoking and obesity (or body weight) is very complex. Although nicotine exposure increases energy expenditure^54^ and suppresses appetite,^55^ heavy smokers seem to have a higher body weight than light smokers,^56^ and there is a tendency of clustering of smoking, obesity, and lower socioeconomic status.^57^ Additionally, smoking increases insulin resistance and is associated with the central fat accumulation that is associated with central obesity and insulin resistance.^58, 59^ Indeed, Wehby *et al.* also suggests that smoking have a heterogeneous effect on body weight.^60^ Nonetheless, in addition to nicotine which reduces body weight, our finding also suggest that acrylamide from smoking might be another component of interest that is associated with body composition alteration. It has been proposed that acrylamide might lower body weight by increasing oxidative stress^26^ that differs from does nicotine. With our findings, it is unable to conclude that the exposure to tobacco smoke or other dietary compounds can fully explain the association observed between acrylamide and body composition. The final effects of smoking, nicotine, acrylamide, obesity, insulin resistance and even behaviors associated with smoking on body composition remain further studies.

Our current study here has demonstrated a negative relationship between HbAA level and body composition in a nationally representative survey of US adults. However, there are several limitations to our study. First, the cross-sectional design does not permit causal inference, and this can only be answered by future longitudinal cohort study. Second, when food is heat-processed, the sugars and lipids within food react with proteins, through the Maillard and other related reactions, to form a wide range of products. These reaction products include advanced glycation/lipoxidation end products, acrylamide and heterocyclic amines, all of which could impact human health and cause diseases. Furthermore, some Maillard reaction products can alter the growth of colonic bacteria, and the thermally-induced modifications of dietary protein can affect allergenicity.^61^ Third, it is possible that the acrylamide exposure only serve as a surrogate of exposure to other chemicals in smokers rather than acrylamide itself functions as an effector. Fourth, our study population is mainly composed of adults, and we cannot extrapolate that the same associations will hold true in children. Lastly, we were not able to acquire information regarding amino acid intake that is known to affect glutathione homeostasis and its detoxification effect.^62^ If the associations reported here can be reproduced in future independent studies, works to identify the underlying mechanisms and effects of long term, but low-dose acrylamide exposure to health outcomes in human are extremely necessary.

In conclusion, here we present the first report identifying a negative association between HbAA level and body composition in a nationally representative survey of US adults. Since acrylamide exposure from food and smoking has become a worldwide concern, further longitudinal clinical and *in vitro* or *in vivo* studies are urgently warranted to elucidate this putative causal relationship.

## Figures and Tables

**Table 1:** Basic demographics of the sample subjects including means (95% CI) of acrylamide adducts and glycidamide adducts concentrations

	*Unweighted no. (%)*	*HbAA (pmol/g Hb)*	P *value between groups*	*HbGA (pmol/g Hb)*	P *value between groups*
Overall	3623 (100)	60.48 (59.32–61.65)		55.64 (54.40–56.92)	
Age, y			<0.001		<0.001
20–39	1251 (34.5)	66.56 (64.35–68.84)		64.45 (62.10–66.89)	
40–59	1055 (29.1)	66.64 (64.14–69.23)		59.34 (56.85–61.94)	
⩾60	1317 (36.4)	51.08 (49.74–52.47)		45.97 (44.34–47.65)	
Gender			<0.001		0.676
Men	1753 (48.4)	64.62 (62.71–66.57)		55.37 (53.53–57.27)	
Women	1870 (51.6)	55.84 (55.47–58.23)		55.90 (54.23–57.63)	
Race			<0.001		<0.001
Mexican American	764 (21.1)	59.98 (58.02–62.01)		61.24 (58.70–63.89)	
Other Hispanic	109 (3.0)	44.96 (40.45–49.98)		48.26 (43.30–53.78)	
Non-Hispanic White	1941 (53.6)	60.56 (58.97–62.20)		56.47 (54.77–58.22)	
Non-Hispanic Black	653 (18.0)	66.69 (63.32–70.24)		51.70 (48.62–54.98)	
Others	156 (4.3)	50.52 (46.15–55.31)		43.56 (38.46–49.35)	
Education levels			<0.001		<0.001
<High school	529 (14.6)	56.64 (54.17–59.22)		53.01 (50.02–56.19)	
High school	1440 (39.7)	66.21 (64.03–68.46)		60.05 (57.85–62.34)	
>High school	1654 (45.7)	57.07 (55.58–58.60)		52.88 (51.20–54.62)	
Annual household income			0.363		0.678
<$25000	1264 (34.9)	61.15 (59.07–63.31)		54.97 (52.79–57.24)	
$25000–55000	1246 (34.4)	60.92 (58.99–62.92)		56.33 (54.21–58.53)	
>$55000	1113 (30.7)	59.23 (57.33–61.19)		55.65 (53.53–57.85)	
BMI, kg/m^2^			<0.001		0.564
<25	1148 (33.4)	66.26 (63.83–68.78)		56.55 (54.15–59.05)	
25–30	1289 (35.1)	59.40 (57.58–61.28)		54.86 (52.85–56.95)	
⩾30	1186 (31.4)	56.45 (54.73–58.22)		55.63 (53.59–57.75)	
Smoking			< 0.001		< 0.001
Nonexposed	769 (21.2)	46.81 (45.67–47.99)		47.74 (45.83–49.72)	
Expose to environmental tobacco smoke	1838 (50.7)	48.39 (47.52–49.27)		45.97 (44.64–47.33)	
Active smoker	1016 (28.1)	109.87 (105.84)		88.28 (84.72–91.98)	
Caffeine intake(mg/day)			<0.001		<0.001
<101	1624 (50.2)	54.17 (52.77–55.61)		51.54 (49.84–53.29)	
⩾101	1608 (49.8)	66.23 (64.28–68.24)		60.89 (58.93–62.92)	
Total saturated fatty acids intake (gm/day)			<0.001		<0.001
<23.33	1616 (50.0)	56.27 (54.78–57.79)		52.30 (50.57–54.10)	
⩾23.33	1616 (50.0)	63.70 (61.84–65.63)		59.95 (58.03–61.94)	

**Table 2:** Linear regression coefficients with one unit increase in log acrylamide adducts and glycidamide adducts concentrations in adults, with results weighted for sampling strategy

	*Unweighted no/Population size*	*Log HbAA (pmol/g Hb)*	P	*Log HbGA (pmol/g Hb)*	P
*Body measures*					
*Body weight* (Kg)					
Model 1	3623/159301526	−3.64±0.94	0.002	0.18±0.65	0.782
Model 2	1891/93731460	−4.71±0.93	<0.001	0.57±0.82	0.497
*Body mass index* (kg/m^2^)					
Model 1	3623/159301526	−1.15±0.24	<0.001	0.13±0.18	0.495
Model 2	1891/93731460	−1.46±0.31	<0.001	0.38±0.29	0.217
*Waist* (cm)					
Model 1	3521/155489162	−2.41±0.65	0.002	0.38±0.52	0.473
Model 2	1876/93206296	−3.72±0.82	<0.001	0.58±0.71	0.429
*Subscapular Skinfold* (mm)					
Model 1	2872/127959324	−1.32±0.29	<0.001	0.28±0.23	0.245
Model 2	1529/77506833	−1.64±0.59	0.015	0.40±0.49	0.427
*Triceps Skinfold* (mm)					
Model 1	3194/140995299	−1.16±0.25	<0.001	0.32±0.20	0.124
Model 2	1698/84636772	−1.34±0.41	0.005	0.45±0.34	0.201
					
*Bioelectrical Impedance Analysis*					
*Estimated fat-free mass* (kg)					
Model 1	1328/75323566	−1.49±0.50	0.009	−0.30±0.43	0.502
Model 2	804/48907658	−1.62±0.38	0.001	−0.65±0.40	0.125
*Estimated fat mass* (kg)					
Model 1	1328/75323566	−1.66±0.55	0.009	0.21±0.44	0.642
Model 2	804/48907658	−3.20±1.05	0.008	−0.14±0.80	0.866
*Estimated percent body fat* (%)					
Model 1	1328/75323566	−0.83±0.44	0.079	0.38±0.52	0.397
Model 2	804/48907658	−1.89±0.77	0.027	0.19±0.65	0.766
*Estimated total body water* (L)					
Model 1	1328/75323566	−1.09±0.36	0.009	−0.21±0.32	0.512
Model 2	804/48907658	−0.65±0.40	0.001	−0.47±0.29	0.127
					
*Dual Energy X-ray Absorptiometry*					
*Android fat mass* (gm)					
Model 1	2896/134154476	−197.79±45.71	0.001	36.40±37.06	0.342
Model 2	1601/82831918	−306.03±80.24	0.002	8.24±68.00	0.905
*Android lean mass* (gm)					
Model 1	2896/134154476	−25.57±33.88	0.462	63.58±27.47	0.035
Model 2	1601/82831918	−101.48±36.21	0.013	19.86±31.73	0.541
*Android percent fat* (%)					
Model 1	2896/134154476	−1.79±0.27	<0.001	0.12±0.26	0.656
Model 2	1601/82831918	−2.45±0.59	0.001	0.16±0.56	0.775
*Gynoid fat mass* (gm)					
Model 1	2896/134154476	−247.77±61.86	0.001	22.02±49.32	0.662
Model 2	1601/82831918	−337.41±95.52	0.003	−3.26±86.49	0.970
*Gynoid lean mass* (gm)					
Model 1	2896/134154476	−258.40±58.55	0.001	−55.83±43.36	0.217
Model 2	1601/82831918	−250.09±64.40	0.001	−62.87±52.30	0.248
*Gynoid percent fat* (%)					
Model 1	2896/134154476	−0.57±0.20	0.012	0.23±0.20	0.274
Model 2	1601/82831918	−0.96±0.36	0.018	0.08±0.34	0.810
*Android to Gynoid ratio*					
Model 1	2896/134154476	−0.04±0.01	<0.001	0.004±0.004	0.281
Model 2	1601/82831918	−0.05±0.01	0.001	0.003±0.006	0.708

Model 1 was adjusted for age, gender, race/ethnicity; model 2 was adjusted for model 1 plus education level, household income, smoking status, caffeine intake, total energy intake, total protein intake, total sugar intake, total carbohydrates intake, total saturated fatty acids intake, metabolic equivalent intensity level for activity.

**Table 3:** Adjusted body measure parameters (S.E.) across quartiles of acrylamide adducts and glycidamide adducts concentrations in adults, with results weighted for sampling strategy

	*Body weight (Kg)*	P *Value*	*BMI (kg/m*^*2*^)	P *Value*	*Estimated fat mass (kg)*	P *Value*	*Android fat mass (gm)*	P *Value*
*HbAA (pmol/g Hb)*								
⩽40.90 (lowest)	82.01 (1.49)		29.13 (0.47)		27.54 (1.45)		2568.76 (109.36)	
⩽53.40	81.72 (1.04)	0.859	29.23 (0.35)	0.831	24.38 (0.87)	0.122	2438.53 (75.43)	0.277
⩽80.10	78.94 (1.10)	0.040	28.32 (0.42)	0.082	23.87 (0.93)	0.064	2327.30 (109.84)	0.084
>80.10 (highest)	76.61 (1.34)	0.005	27.60 (0.40)	0.003	21.90 (1.02)	0.008	2167.97 (86.02)	0.002
*P for trend*	0.002		0.005		0.035		0.003	

*HbGA (pmol/g Hb)*								
⩽39.50(lowest)	78.57 (1.45)		27.94 (0.45)		24.95 (1.10)		2332.83 (100.06)	
⩽55.40	79.55 (1.15)	0.393	28.41 (0.41)	0.228	24.94 (1.02)	0.991	2360.12 (94.60)	0.774
⩽81.30	79.80 (1.21)	0.533	28.67 (0.44)	0.276	23.84 (1.06)	0.540	2440.82 (107.47)	0.641
>81.30 (highest)	80.88 (1.45)	0.170	29.07 (0.47)	0.053	24.24 (0.87)	0.579	2394.51 (82.39)	0.558
*P for trend*	0.606		0.306		0.864		0.950	

Adjusted for full model.

**Table 4:** Linear regression coefficients (s.e.) between log acrylamide adducts, BMI, Estimated fat mass in different subpopulations of sample subjects with results weighted for sampling strategy

	*BMI(kg/m*^*2*^)		*Android fat mass (gm)*	
	*βcoeff (s.e.)*	P *value*	*βcoeff (s.e.)*	P *value*
*Age, y*				
20–39	−2.22 (0.44)	<0.001	−417.50 (104.16)	0.001
⩾40	−1.14 (0.50)	0.037	−282.63 (113.94)	0.025
				
*Gender*				
Men	−1.54 (0.40)	0.002	−323.54 (81.37)	0.001
Women	−1.06 (0.71)	0.157	−228.69 (136.28)	0.114
				
*Race*				
White	−1.33 (0.39)	0.004	−367.87 (100.50)	0.002
Others	−2.00 (0.67)	0.009	−169.63 (119.67)	0.177
				
*Education levels*				
⩽High school	−2.15 (0.42)	<0.001	−426.71 (105.61)	0.001
>High school	−0.97 (0.45)	0.046	−252.66 (107.54)	0.033
				
*Smoking*				
Nonexposed	−0.17 (1.06)	0.874	−74.31 (256.57)	0.777
Expose to environmental tobacco smoke	−1.39 (0.57)	0.027	−272.34 (122.78)	0.042
Active smoker	−1.49 (0.33)	<0.001	−355.23 (71.47)	<0.001
				
*BMI*				
<28	−0.52 (0.22)	0.032	−160.01 (50.30)	0.006
⩾28	−0.49 (0.36)	0.193	−148.35 (107.99)	0.190

Abbreviation: BMI; body mass index.

Adjusted for full model.

**Table 5:** Linear regression coefficients (SE) between log acrylamide adducts, BMI, Estimated fat mass in active smokers across dichotomy of serum cotinine level with results weighted for sampling strategy

*Cotinine (ng/ml)*	*⩽178*			*>178*		
	*Unweighted no/Population size*	*Log HbAA (pmol/g Hb)*	P	*Unweighted no/Population size*	*Log HbAA (pmol/g Hb)*	P
*Body measures*						
Body weight (Kg)	240/13102823	−4.00 (2.72)	0.162	208/11642194	−7.33 (2.63)	0.015
Body mass index (kg/m^2^)	240/13102823	−1.28 (0.80)	0.128	208/11642194	−2.06 (0.83)	0.026
Waist (cm)	239/13073240	−3.78 (1.72)	0.044	207/11548150	−5.12 (1.56)	0.005
Subscapular Skinfold (mm)	194/10679965	−2.53 (1.07)	0.033	172/9870941	−3.12 (0.49)	<0.001
Triceps Skinfold (mm)	218/11961446	−2.74 (0.86)	0.006	199/11219535	−1.72 (0.53)	0.005
						
*Bioelectrical Impedance Analysis*						
Estimated fat-free mass (kg)	135/7693204	−1.12 (2.08)	0.600	125/7249712	−2.73 (2.48)	0.294
Estimated fat mass (kg)	135/7693204	−3.44 (1.75)	0.068	125/7249712	−2.89 (0.95)	0.011
Estimated percent body fat (%)	135/7693204	−2.71 (1.15)	0.032	125/7249712	−1.31 (1.06)	0.240
Estimated total body water (L)	135/7693204	−0.83 (1.53)	0.597	125/7249712	−1.94 (1.81)	0.306
						
*Dual Energy X-ray Absorptiometry*						
Android fat mass (gm)	211/11556404	−423.27 (127.76)	0.005	190/10697719	−340.29 (136.49)	0.026
Android lean mass (gm)	211/11556404	−154.61 (113.96)	0.195	190/10697719	−167.40 (138.45)	0.247
Android percent fat (%)	211/11556404	−3.30 (1.00)	0.005	190/10697719	−2.90 (0.0.76)	0.002
Gynoid fat mass (gm)	211/11556404	−484.40 (181.41)	0.017	190/10697719	−421.32 (197.66)	0.051
Gynoid lean mass (gm)	211/11556404	−188.05 (213.10)	0.391	190/10697719	−623.60 (275.44)	0.040
Gynoid percent fat (%)	211/11556404	−2.05 (0.87)	0.033	190/10697719	−0.55 (0.523)	0.315
Android to Gynoid ratio	211/11556404	−0.04 (0.02)	0.039	190/10697719	−0.07 (0.02)	0.001

Abbreviation: BMI; body mass index.

Adjusted for full model.
